# Bone turnover markers are correlated with total skeletal uptake of ^99m^Tc-methylene diphosphonate (^99m^Tc-MDP)

**DOI:** 10.1186/1756-6649-9-3

**Published:** 2009-03-30

**Authors:** Janaka Lenora, Kristina Norrgren, Ola Thorsson, Per Wollmer, Karl J Obrant, Kaisa K Ivaska

**Affiliations:** 1 Department of Orthopaedics, Malmö University Hospital, Lund University, SE 20502 Malmö, Sweden; 2 Department of Clinical Physiology, Malmö University Hospital, Lund University, SE 20502 Malmö, Sweden

## Abstract

**Background:**

Skeletal uptake of ^99m^Tc labelled methylene diphosphonate (^99m^Tc-MDP) is used for producing images of pathological bone uptake due to its incorporation to the sites of active bone turnover. This study was done to validate bone turnover markers using total skeletal uptake (TSU) of ^99m^Tc-MDP.

**Methods:**

22 postmenopausal women (52–80 years) volunteered to participate. Scintigraphy was performed by injecting 520 MBq of ^99m^Tc-MDP and taking whole body images after 3 minutes, and 5 hours. TSU was calculated from these two images by taking into account the urinary loss and soft tissue uptake. Bone turnover markers used were bone specific alkaline phosphatase (S-Bone ALP), three different assays for serum osteocalcin (OC), tartrate resistant acid phosphatase 5b (S-TRACP5b), serum C-terminal cross-linked telopeptides of type I collagen (S-CTX-I) and three assays for urinary osteocalcin (U-OC).

**Results:**

The median TSU of ^99m^Tc-MDP was 23% of the administered activity. All bone turnover markers were significantly correlated with TSU with r-values from 0.52 (p = 0.013) to 0.90 (p < 0.001). The two resorption markers had numerically higher correlations (S-TRACP5b r = 0.90, S-CTX-I r = 0.80) than the formation markers (S-Total OC r = 0.72, S-Bone ALP r = 0.66), but the difference was not statistically significant. TSU did not correlate with age, weight, body mass index or bone mineral density.

**Conclusion:**

In conclusion, bone turnover markers are strongly correlated with total skeletal uptake of ^99m^Tc-MDP. There were no significant differences in correlations for bone formation and resorption markers. This should be due to the coupling between formation and resorption.

## Background

Bone metabolism can be assessed by biochemical means using bone turnover markers (BTM) measured in serum or urine [1]. BTMs can be used in the monitoring of antiresorptive therapy [2,3] and there is increasing evidence that at least some BTMs can be predictive for bone loss [4] and fracture [5,6]. They are, however, also subjected to rapid changes due to reasons other than bone metabolism [7], such as diurnal variation, other tissue damages and food intake [8]. Some of the BTMs reflect bone formation, while others are associated to bone resorption. However, both formation and resorption markers are usually affected by changes in turnover due to the coupling between these two processes [1].

Several attempts have been made to assess the skeletal metabolic activity by using skeletal uptake of radiolabelled, bone seeking, substances. Bisphosphonates, structurally similar to the inorganic pyrophosphates in bone matrix, have high affinity to bind to bone mineral [9]. Especially, they bind to the exposed sites that undergo high bone turnover. Technetium-99m (^99m^Tc) labelled diphosphonates are commonly used in scintigraphic uptake studies to detect lesions in conditions such as cancer metastasis, occult fractures and osteomyelitis due to their high affinity to metabolically active sites in bone. In these procedures the skeletal or extra-osseous accumulation of ^99m^Tc labelled methylene diphosphonate (^99m^Tc-MDP) is used to identify the lesions as "hot spots" [10,11]. In earlier studies the measurement of 24-hour whole body retention of ^99m^Tc-MDP was used to assess the skeletal metabolism [12,13], before introducing the regional quantification of ^99m^Tc-MDP activity by D'Addabbo et al [14] and Brenner et al [15]. These techniques have been found to be useful techniques for estimating skeletal turnover rate at the time of the measurement. The regional quantification after 5-hours has the advantage over 24-hour retention that it directly gives a measure of skeletal uptake and a shorter time period is needed. To the best of our knowledge, the correlation between bone metabolism assessed by skeletal uptake of ^99m^Tc-MDP and by bone turnover markers has been evaluated only in a few studies [13,16-18].

This study was designed to assess the correlation between the skeletal uptake of ^99m^Tc-MDP, and nine bone turnover markers including markers of bone formation and bone resorption and urinary osteocalcin. Our aim was to elucidate if markers reflect total skeletal turnover determined by skeletal uptake of ^99m^Tc-MDP. Furthermore, we aimed to investigate if uptake of ^99m^Tc-MDP is more related to bone formation or resorption, assuming that if any of the bone formation markers had a significantly greater correlation with TSU, over the others; it could have been regarded as a relatively specific measure of bone formation.

## Methods

### Participating women

22 postmenopausal women who had sought medical advice or treatment for minor orthopaedic complains (such as non-fracture trauma, back pain, vertebral fractures, ankle fractures) at least 6 months before the recruitment and who had never been treated with bisphosphonates were selected from the registers of the orthopaedic clinic at Malmö University Hospital. Patients with primary hyperparathyroidism, hyperthyroidism, osteomalacia, chronic malnutrition, any malignancy, hepatic cirrhosis, joint prosthesis; or patients who had been treated with systemic estrogens, therapeutic calcium, vitamin D or corticosteroids within the last one year were not included. When the study was started, they were free from the condition that had originally brought them to the clinic. Fractures within 2 years prior to the study were also recorded.

### Bone mineral density

Areal bone mineral density (BMD) of total body, lumbar spine, femoral neck and bone mineral content (BMC) of the total body were measured by dual-energy x-ray absorptiometry (DXA) (Lunar DPX-L^® ^Madison, USA). The precision of measurements assessed by duplicate measurements on 15 elderly women after repositioning were 0.5% for total body BMD, 1.3% for total body BMC, 1.2% for lumber spine BMD, and 3.9% for femoral neck BMD.

### Bone scintigraphy

Bone scintigraphy procedure was performed within 28 days after the DXA scanning according to a method described by Brenner et al [15]. An injection of 520 (517 ± 15) MBq of ^99m^Tc-MDP (Medronate^®^, Amersham international) was given. The radio activity was measured in the syringe both before and after injection to enable an accurate determination of injected activity. Whole body imaging was performed directly (3 minutes) after injection and 5 hours after injection. A double-headed gamma camera system (Siemens Multispect 2) equipped with low energy high-resolution collimators was used for the scan. The scan speed was 40 cm/min for the image at 3 minutes and 15 cm/min for the image after 5 hours. The images were stored in a 1024 × 256 matrix for image processing.

**Figure 1:**
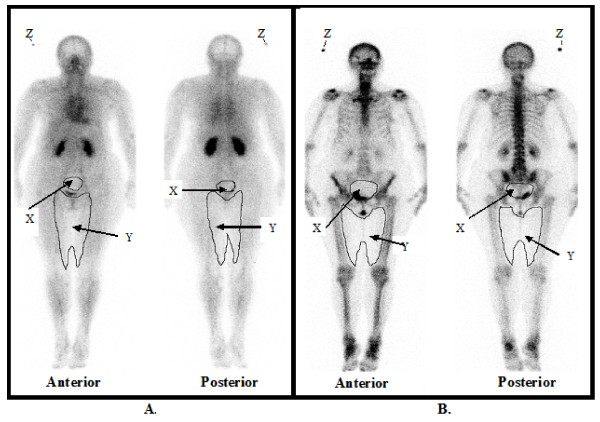
**Whole body scan images of one of the study participants at 3 minutes (A) and at 5 hours (B)**. Regions of interest (ROI) were drawn in the anterior and posterior images to quantify the activity in whole body, urinary bladder (X), and the adductor muscles of both thighs (Y), as described by Brenner *et al.*[15] Z = area marked for counting the background radiation.

Regions of interest (ROI) were drawn in the anterior and posterior images to quantify the activity in whole body, urinary bladder, and the adductor muscles of both thighs, (Figure 1) as described by Brenner *et al.*[15]. The geometric mean of the anterior and posterior image was used in the calculation of activity content and the 3-minute image was used as a reference to calculate the percentage uptake in the later image. For all data the numbers of counts in the regions were corrected for decay of ^99m^Tc. The soft tissue activity was calculated from the adductor compartment of both thighs as follows: activity of adductor muscles at 5 hours divided by the activity of adductor muscles at 3 minutes and multiplied by whole body activity at 3 minutes. All activity was considered to be excreted from the body, only via urine. The excretion was calculated from the difference in whole body activity between two imaging times. Correction for radioactive decay and scan speed was done. The total skeletal uptake (TSU) of ^99m^Tc-MDP was calculated as (whole body radioactivity at 3 min – urinary excretion – soft tissue uptake at 5 hour)/whole body radioactivity at 3 min × 100% [15].

### Serum and urine samples for bone turnover markers

Non-fasting blood samples were collected before the scintigraphy procedure (at 9.00 am). Serum was separated within 2 hours after phlebotomy. Non-fasting urine samples were collected at 9.00 am. Serum and urine samples were stored at -80°C for the analysis of bone turnover markers.

### Bone turnover markers

All the analyses were done at the same time. Bone-specific alkaline phosphatase (S-Bone ALP) was determined by using Metra BAP immunoassay (Quidel Corporation), with an intra- and inter- assay CV of 3.6% and 4.4%, respectively. Serum intact osteocalcin [S-OC(1–49)], serum total osteocalcin (S-Total OC) and serum total gamma-carboxylated osteocalcin (S-cOC) were determined by previously described, in-house protocols with intra- and inter- assay CV of less than 5% and 8%, respectively, for all the assays [19].

Serum C-terminal cross-linking telopeptides of type I collagen (S-CTX-I) were determined by Elecsys β-Cross Laps^® ^immunoassay (Roche diagnostics) with intra- and inter- assay CV of 5.9% and 5.8%, respectively. Serum tartrate-resistant acid phosphatase 5b (S-TRACP5b) was assessed by a solid phase, immunofixed, enzyme activity assay as described earlier [20] with an intra- and inter- assay CV of 1.8% and 2.2%, respectively.

Three different assays of urinary osteocalcin, total osteocalcin (U-TotalOC), long osteocalcin (U-LongOC) and mid osteocalcin (U-MidOC) were analyzed as previously published with intra- and inter- assay CVs of 14% and < 27% (U-TotalOC), 4.3% and < 14% (U-LongOC), and 1.7% and < 12% (U-MidOC), respectively [21].

Urinary creatinine was measured by the kinetic Jaffe reaction with a Beckman synchron LX20-4, with CVs of 3% or less. All the measurements of urinary osteocalcin were corrected for urinary creatinine and expressed as ratios.

### Statistical analysis

Statistica for Windows (version 7.1, Stat Soft Inc) software was used for the statistical analysis. The results were expressed as median and inter quartile range (IQR). The correlations of bone turnover markers and the total skeletal uptake of ^99m^Tc-MDP were assessed by using Spearman rank correlations. Group comparisons were done using Mann-Whitney U test. P-values less than 0.05 were considered statistically significant.

### Ethics

All steps of the study were approved by the ethical review committee, Lund University, Sweden in accordance with the Declaration of Helsinki. Informed, consent was obtained from each of the participants prior to the study.

## Results

### Basic characteristics

The median age of the women was 65 years (range 52–80). The median total body BMD was 1.02 g/cm^2 ^(IQR 0.97 – 1.08) (Table 1). Eight women had osteoporosis, defined as T score ≤ -2.5 at spine (n = 7) or at femoral neck (n = 1). Eight women had sustained a fracture within 2 years (range 0.5 – 2) before the study, including vertebral compression fractures (n = 6), distal radius fracture (n = 1) and ankle fracture (n = 1). Of them, five women had osteoporosis based on lumbar spine or femoral neck T-score.

**Table 1:** Baseline characteristics, scintigraphy results and bone turnover markers of participants (n = 22).

	Median (inter quartile range)	Correlations with TSU of ^99m^Tc-MDP	
		
		r	p
**Anthropometry**			
Age (years)	65 (59 – 73)	0.06	0.79
Height (cm)	162 (158 – 167)	0.03	0.90
Weight (kg)	65 (60 – 79)	-0.08	0.071
BMI (kg/m^2^)	25.4 (22.8 – 29.1)	-0.18	0.43
**Bone mass**			
Total body BMC (g)	2075 (1933 – 2208)	-0.23	0.30
Total body BMD (g/cm^2^)	1.02 (0.97 – 1.08)	-0.36	0.10
**Scintigraphy**			
Total skeletal uptake (%)	22.9 (18.8 – 27.9)	-	-
**Bone Formation markers**			
S-Bone ALP (U/L)	23.0 (17.0 – 28.0)	**0.66**	**< 0.001**
S-Total OC (μg/L)	5.8 (5.0 – 8.5)	**0.72**	**< 0.001**
S-OC(1–49) (μg/L)	3.0 (2.2 – 5.0)	**0.65**	**< 0.01**
S-cOC (μg/L)	6.9 (5.4 – 9.1)	**0.67**	**< 0.001**
**Bone resorption markers**			
S-TRACP5b (U/L)	3.1 (2.5 – 3.9)	**0.90**	**< 0.001**
S-CTX-I (ng/L)	228 (173 – 326)	**0.80**	**< 0.001**
**Urine osteocalcin**			
U-Total OC/crea (μg/nmol)	28.3 (19.7 – 32.0)	**0.52**	**0.013**
U-Mid OC/crea (μg/nmol)	1.3 (0.9 – 2.0)	**0.76**	**< 0.001**
U-Long OC/crea (μg/nmol)	0.02 (0.01 – 0.04)	**0.72**	**< 0.001**

Results are shown as median values (interquartile range). Spearman rank correlations (r values) to total skeletal uptake (TSU) of ^99m^Tc-MDP are also given.

### Scintigraphy

The median value for total skeletal uptake of ^99m^Tc-MDP at 5 hours was 23% (IQR (18.8 – 27.9). There were no statistically significant associations between total skeletal uptake and total body BMD, total body BMC, body weight, BMI or age (Table 1).

### Bone turnover markers

**Figure 2:**
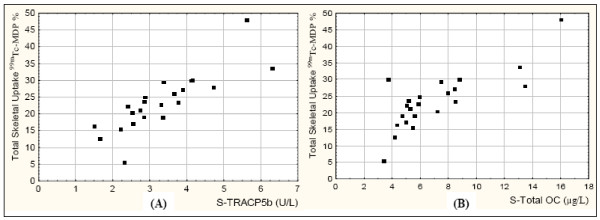
**The association of total skeletal uptake (TSU) of ^99m^Tc-MDP with (A) bone resorption marker, S-TRACP5b, r_(spearman) _= 0.90, p < 0.001 and (B) bone formation marker, S-Total OC, r_(spearman) _= 0.72, p < 0.001**.

All the bone turnover markers were highly correlated with total skeletal uptake of ^99m^Tc-MDP with r-values from 0.52 for U-TotalOC (p = 0.013) to 0.90 for S-TRACP5b (p < 0.001) (Table 1 and Figure 2). The two resorption markers had numerically higher correlations (S-TRACP5b r = 0.90, S-CTX-I r = 0.80) than the formation markers (S-Total OC r = 0.72, S-Bone ALP r = 0.66), but the difference was not statistically significant.

### Comparison of women with and without a recent fracture

We also compared women who had sustained a fracture two years prior to the study (n = 8) to the other women (n = 14). Women with a recent fracture had lower total body BMD, higher TSU of ^99m^Tc-MDP, and higher levels of bone formation markers than women without a recent fracture (Table 2). There were no significant differences in anthropometry, BMC, bone resorption markers or U-OCs, although the levels of resorption markers seemed to be also slightly elevated in the fracture group.

**Table 2:** Baseline characteristics, BMD, scintigraphy results and bone turnover markers in women with and without a recent fracture.

	Women with a recent fracture (n = 8)	Women without a recent fracture (n = 14)	p
**Anthropometry**			
Age (years)	62 (58 – 71)	66 (60 – 73)	0.54
Height (cm)	163 (159 – 168)	162 (157 – 167)	0.68
Weight (kg)	64 (61 – 69)	68 (57 – 84)	0.81
BMI (kg/m^2^)	24.2 (22.6 – 27.5)	26.5 (22.8 – 29.8)	0.41
			
**Bone mass**			
Total body BMC (g)	1976 (1929 – 2051)	2127 (1960 – 2247)	0.207
Total body BMD (g/cm^2^)	0.97 (0.94 – 1.04)	1.04 (1.00 – 1.08)	**0.041**
			
**Scintigraphy**			
Total skeletal uptake (%)	28.6 (21.3 – 31.8)	21.6 (17.0 – 24.8)	**0.048**
			
**Bone formation Markers**			
S-Bone ALP (U/L)	27.0 (24.0 – 34.5)	21.0 (16.0 – 25.0)	**0.034**
S-Total OC (μg/L)	8.2 (5.5 – 13.3)	5.8 (4.4 – 7.3)	**0.020**
S-OC(1–49) (μg/L)	4.9 (3.0 – 10.0)	2.9 (2.1 – 4.4)	**0.031**
S-cOC (μg/L)	10.2 (6.6 – 16.5)	6.1 (5.3 – 7.8)	**0.017**
			
**Bone resorption Markers**			
S-TRACP5b (U/L)	3.8 (2.9 – 5.2)	2.8 (2.4 – 3.7)	0.076
S-CTX-I (ng/L)	411 (186 – 639)	203 (173 – 253)	0.066
			
**Urine Osteocalcin**			
U-Total OC/crea (μg/nmol)	30.5 (27.0 – 32.1)	25.5 (17.4 – 29.8)	0.13
U-Mid OC/crea (μg/nmol)	1.9 (1.3 – 2.4)	1.1 (0.9 – 1.7)	0.12
U-Long OC/crea (μg/nmol)	0.03 (0.01 – 0.08)	0.02 (0.01 – 0.03)	0.41

Results are shown as median values (inter quartile range). P values for Mann-Whitney U test are also given.

### Comparison of women with and without osteoporosis

There was no statistically significant difference in bone turnover markers or in total skeletal uptake of ^99m^Tc-MDP between women with osteoporosis (n = 8) and other women (n = 14) (data not shown).

## Discussion

We have studied the association between nine bone turnover markers, representing different aspects of bone turnover, and total skeletal metabolism, as assessed by scintigraphic measurement of total skeletal uptake of ^99m^Tc-MDP. All bone turnover markers were highly correlated to bone metabolism assessed by total skeletal uptake of ^99m^Tc-MDP.

S-TRACP5b and S-CTX-I, the markers of bone resorption, were found to be numerically best correlated with TSU of ^99m^Tc-MDP. The correlations for bone formation markers were, however, also highly significant and it was not evident which of the bone turnover markers were associated to total skeletal metabolism the most. Studies with ^99m^Tc-MDP suggest that MDP uptake reflects a combination of skeletal blood flow and osteoblastic activity [22,23]. However, markers of bone formation not seemed to be more correlated with such uptake than markers of bone resorption. The lack of difference between formation and resorption markers could be due to the coupling of these two processes. Moreover, studies with radio-labelled bisphosphonates have shown that bisphosphonates localize to regions where new bone is being deposited and newly formed crystals provide a surface area of exposed mineral available to adsorb bisphosphonates, but are also incorporated where osteoclasts are resorbing bone [24].

In addition, the precision and accuracy of the assays for bone turnover markers differ. These differences in assay performance may have influenced the correlations between bone markers and TSU of ^99m^Tc-MDP, making the comparison of markers more difficult.

The highest r-value (0.90) was observed for S-TRACP5b. TRACP5b is an enzyme produced by bone-resorbing osteoclasts and the activity of TRACP5b in serum reflects the number of active osteoclasts [25,26]. The r-value for S-CTX-I was almost as high (0.80) as for S-TRACP5b. CTX-I results from cathepsin K-mediated degradation of type I collagen by osteoclasts [27]. The number of bone-resorbing osteoclasts (TRACP5b), as well as the amount of degraded type I collagen (CTX-I) should be tightly correlated to the rate of skeletal metabolism. The collection of samples at non-fasting status may, however, have interfered with the correlation for CTX-I, as it's levels are known to be influenced by food intake [8].

The r-values for formation markers S-OC and S-bone ALP were slightly lower (0.65 – 0.72). S-OC has a short half-life in circulation [28] and it may be more susceptible to preanalytical variability, such as *in vitro *degradation [29]. Moreover, circulating OC may contain molecules derived from both formation and resorption processes [30]. Bone-specific alkaline phosphatase is an enzyme originating from osteoblasts and needed in osteoid formation and mineralization. Although the methods currently available detect preferentially the bone-specific isoform of the enzyme, they still show a certain degree of cross-reactivity between bone and liver isoforms [31]. The r-values for two of the three urinary OC assays were of similar magnitude (0.72 and 0.76) than for serum OC (0.65, 0.67 and 0.72).

Previous studies on healthy individuals, individuals with endocrine disorders (such as Cushing's syndrome, thyrotoxicosis and primary hyperparathyroidism) or other skeletal diseases (such as heterotrophic pulmonary ostoarthropathy) have shown TSU of ^99m^Tc-MDP to be correlated to conventional bone turnover markers such as osteocalcin, urinary deoxypyridinoline [18], total alkaline phosphatase, and urinary hydroxyproline [13,16-18] but data on many currently available, more specific and sensitive bone turnover markers has been lacking.

We did not detect correlation for TSU and age or for TSU and BMD. Previous studies on healthy women, have shown that the TSU of ^99m^Tc-MDP is positively correlated with age (n = 40, 84) [16,32] and negatively correlated with BMD (n = 86) [33]. The absence of such correlation in our study may be due to limited sample size.

When the women who had sustained fractures within two years prior to the study were compared to the others, women with recent fracture had higher level of bone formation markers and higher level of TSU of ^99m^Tc-MDP. This is in line with our previous findings that bone formation markers remains elevated up to 1–2 years after fracture [34,35]. Only one out of eight women had visible focal uptake on the scintigram, on the site of prior fracture. Most probably the increase of bone turnover in the fractured individuals is due to the generalized post traumatic skeletal process taking place after fracture [36] as well local increase at the fracture site.

A main strength of this study is that we analyzed several BTMs reflecting different aspects of bone metabolism. In particular, the novel bone turnover markers such as S-TRACP5b and urinary osteocalcins have not been evaluated by using TSU of ^99m^Tc-MDP in any of the earlier studies. There are also limitations. Small sample size hindered us to compare which of the BTMs that correlated most with TSU of ^99m^Tc-MDP. This should be possible with larger sample sizes, including also samples for relatively high and low levels of formation and resorption, such as children, and patients on anabolic treatments (high bone formation rate), patients with osteolytic bone metastases (high bone resorption rate) or patients on anti-resorptive therapy (low bone formation and resorption rate). Another limitation was with the scanning of scintigraphic procedure used. When we take the whole body image at 3 min, with the speed of 40 cm/min, it took about 3 minutes for the camera to reach the thighs where soft tissue uptake was calculated. It was assumed that 100% of radioisotope is in soft tissue at this early image, but by this time (approximately 6 minutes) some of radioisotope could have already entered the skeleton or filtered by the kidneys. When the study was initiated, information on the effect of feeding on BTMs was not available. Samples were collected without fasting and the non-fasting status may have had minor influence on the results of a few markers, in particular S-CTX-I [8]

## Conclusion

In conclusion, biochemical markers of bone turnover are strongly correlated with the skeletal metabolism as measured by TSU of ^99m^Tc-MDP. Although ^99m^Tc-MDP uptake is largely driven by osteoblastic activity, there were no significant differences in correlations between skeletal uptake of ^99m^Tc-MDP and bone formation markers or bone resorption markers. This could be due to coupling between formation and resorption.

## Abbreviations

^99m^Tc-MDP: Technitum 99m labelled methyline diphosphonate; TSU: Total skeletal uptake; BMD: Areal bone mineral density; BMC: Bone mineral content; DXA: Dual energy x-ray absorptiometry; IQR: Inter quartile range; S-Bone ALP: Serum bone specific alkaline phosphatise; S-Total OC: Serum total osteocalcin; S-OC(1–49): Serum intact osteocalcin; S-cOC: Serum carboxylated osteocalcin; S-TRACP5b: Serum tartrate resistant acid phosphatase 5b; S-CTX-I: Serum C-terminal cross-linked telopeptides of type I collagen; U-Total OC/crea: Urinary total osteocalcin to urinary creatinine ratio; U-Mid OC/crea: Urinary mid osteocalcin to urinary creatinine ratio; U-Long OC/crea: Urinary long osteocalcin to urinary creatinine ratio.

## Competing interests

The authors declare that they have no competing interests.

## Authors' contributions

JL was responsible for the progress of the study, performed the statistical analysis, interpreted the data and wrote the manuscript. KN and OT performed the scintigraphy procedure, helped to draft the manuscript and helped commenting on the manuscript. PW was involved in planning of the study and helped to draft the manuscript. KJO designed the study, and helped with interpretation of results, and manuscript writing. KKI analysed the bone turnover markers and was involved in interpretation of results and writing of the manuscript. All authors have read and approved the final manuscript.

## Pre-publication history

The pre-publication history for this paper can be accessed here:


